# Impact of school closures during the pandemic on screen time and behavior of children: Evidence from a developing country

**DOI:** 10.1192/j.eurpsy.2022.1768

**Published:** 2022-09-01

**Authors:** P. Malhi, B. Bharti, M. Sidhu

**Affiliations:** 1 Post Graduate Institute of Medical Education and Research, Department Of Pediatrics, Chandigarh, India; 2 MCM DAV College, Department Of Psychology, Chandigarh, India

**Keywords:** pandemic, school closure, screen time, behavior

## Abstract

**Introduction:**

Serious concerns regarding the indirect physical and mental health impact of the extended school closure measure to control the spread of the pandemic have been raised, however, the extent of the problem remains unquantified in India.

**Objectives:**

To examine the impact of school closures on recreational screen time, emotional, and behavioral functioning of school-going children during the pandemic.

**Methods:**

The survey utilized a Google form that was sent to parents of children (6-14 years) through emails and social media platforms. Parents were asked to report on the child’s duration of recreational screen time and whether the child’s overall behavioral functioning had changed since the school closures. The child’s emotional and behavioral functioning was assessed by the Strength and Difficulties Questionnaire (SDQ). The study was cleared by the Ethics committee.

**Results:**

A total of 160 parents were recruited for the study. Overall, a little more one-fourth (28.1%) of the children’s behavior was reported to have worsened. The mean recreational screen time was 2.65 hours (SD=1.89). A significantly higher proportion of children whose behavior worsened after school closures, relative to those whose behavior improved or remained same, had scores in the abnormal range of functioning on three of the subscales of SDQ. Stepwise multiple regression analysis indicated that recreational screen time explained 2% of the variance in the total SDQ score (F=4.18. P=.04).

**Conclusions:**

Increase in psychological services supporting healthy behaviors and anticipatory telehealth consultations for high-risk children and families is the need of the hour to foster psychological wellbeing during the pandemic.

**Disclosure:**

No significant relationships.

